# The Natural History and Risk Factors for the Development of Food Allergies in Children and Adults

**DOI:** 10.1007/s11882-024-01131-3

**Published:** 2024-02-28

**Authors:** Eric C. K. Lee, Brit Trogen, Kathryn Brady, Lara S. Ford, Julie Wang

**Affiliations:** 1 The Children’s Hospital at Westmead, Locked Bag 4001, Westmead, NSW 2145 Australia; 2 Jaffe Food Allergy Institute, Icahn School of Medicine at Mount Sinai, New York, USA; 3 Department of Pediatrics, New York-Presbyterian Hospital/Weill Cornell Medical Center, New York, USA; 4 Sydney Medical School, The University of Sydney, The University of Sydney, NSW 2006 Australia

**Keywords:** Natural history of food allergy, Persistence of food allergy, Adolescent-onset food allergy, Adult-onset food allergy, Cow’s milk allergy, Egg allergy

## Abstract

**Purpose of Review:**

This narrative review explores food allergy prevalence and natural history stratified by life stages, especially in context of evolving knowledge over the last few decades.

**Recent Findings:**

The prevalence of food allergy remains highest in early childhood with common food triggers being cow’s milk, soy, hen’s egg, wheat, peanut, tree nuts, sesame, fish, and shellfish. This correlates with certain risk factors especially pertinent in the postnatal period which appear to predispose an individual to developing a food allergy. Some allergies (such as milk and egg) were previously thought to be easily outgrown in early life; however, recent studies suggest increasing rates of persistence of these allergies into young adulthood; the reason behind this is unknown. Despite this, there is also evidence demonstrating that food allergies can be outgrown in adolescents and adults.

**Summary:**

An understanding of the paradigm shifts in the natural history of food allergy allows clinicians to provide updated, age-appropriate, and tailored advice for patients on the management and prognosis of food allergy.

## Introduction

Food allergy is characterized by an inappropriate immune response upon ingestion of certain foods. While debate is ongoing regarding the underlying etiology of food allergy, the body’s responses to specific allergens are known to evolve over time, with food allergies both emerging and resolving at every life stage. Although the majority of food allergies arise in children, a large proportion of childhood-onset food allergies resolve during school age [1]. Similarly, while new-onset allergies in adulthood are less common, over half of food-allergic adults report at least one food allergy with onset in their adult years [2•]. Both accurate diagnosis of food allergies and evaluation of resolution are important to prevent serious health and socioeconomic impacts.

The prevalence of food allergy has risen dramatically over the past 30 years. Although increased awareness of food allergy may account for some of the increase in reported prevalence, true food allergy in all age groups is believed to be increasing [3]. This increase is thought to be due to complex interactions between genetic and environmental factors including growing adoption of a westernized lifestyle globally, and changes to infant feeding practices in recent decades. These increased rates of food allergy pose challenges at both individual and population health levels. Given the potentially life-threatening nature of anaphylaxis due to accidental ingestion, food-allergic individuals and their families often maintain heightened vigilance for allergen avoidance that has significant social, psychological, and cultural impacts. Dietary restrictions due to food allergy can have serious nutritional impacts. Therefore, as new food allergy prevention and treatment modalities emerge, developing an awareness of the natural course of food allergies can aid in assessing risks and benefits of intervention.

In this review, we will highlight key insights into food allergies at several life stages, focusing on the specific factors impacting children, adolescents, and adults with IgE-mediated food allergies. A better understanding of the natural history of food allergies can enable physicians to maintain an appropriate index of suspicion for their patients, as well as tailor their counseling and advice to specific age groups, improving the management of this widespread condition.

## Infancy and Early Childhood

The onset of food allergies commonly occurs in infancy and early childhood, with prevalence highest in this age group, reported between 8 and 17% [11, 23–26]. The causes have not been fully elucidated although there are many predictors of early development of allergic sensitization and clinical allergy.

### Risk Factors for Developing Food Allergy in Infancy

There is a genetic predisposition for food allergy, with an immediate family history of atopy associated with an increased risk of developing food allergy. In the Australian HealthNuts study, children who had two or more atopic family members were shown to have an increased risk of having food allergy (odds ratio for food allergy of 1.8) [27]. Similar observations were found in a Japanese cohort, where a history of atopy in both parents resulted in an odds ratio of 2.6 [28].

Certain antenatal and perinatal characteristics are associated with early-onset food allergy, suggesting the involvement of both genetic and environmental factors. In utero exposures such as obesity, smoking, and dietary restriction correlate with increased Th2 cytokine signatures in cord blood, and subsequent allergic disease in children. Additionally, perinatal events such as delivery via caesarean section, formula feeding, and exposure to antibiotics and other synthetic chemicals can result in gut dysbiosis [29–32]. Gut dysbiosis is linked to the development of food allergy as it has been shown that certain gut microbiota profiles can negatively influence immune regulation and maturation, and subsequently prime the infant gut towards allergic sensitization and inflammation [30].

Environmental risk factors continue to play a role beyond the perinatal period. There are well-recognized cultural variations in the development of certain food allergies according to local dietary patterns and infant feeding practices [33]. The specific effect of timing and route of food introduction on food allergy is at the core of Lack’s well-recognized “dual allergen exposure” hypothesis, whereby first exposure of food allergens via non-oral routes, especially in the context of an inflammatory setting such as the impaired skin barrier of an infant with atopic dermatitis, results in allergic sensitization [34]. The strong association between infant atopic dermatitis and food allergies has been recognized for at least two decades [35], and more recent mechanistic studies suggest the presence of an inflammatory milieu (including interleukin-25, interleukin-33, and thymic stromal lymphopoietin) at the epithelial interface being a key factor in the development of allergic sensitization [35–38]. Respiratory sensitization with intranasal peanut flour resulting in Th2 cytokine profiles in a mouse model has also been demonstrated [39]. This hypothesis, accompanied by strongly supportive prospective research, underpins recent recommendations for early oral introduction of foods to prevent the development of food allergies, as discussed later.

Another environmental risk factor is vitamin D status: it has been shown that children who live in latitudes further from the equator [40, 41], are born in autumn or winter [42], or have biochemical vitamin D deficiency [43] have higher rates of food allergy, possibly due to vitamin D’s immune regulatory effect [44, 45]. Despite these observations, vitamin D supplementation has not been demonstrated to prevent food allergies [45].

Finally, genetic and environmental factors can interact in poorly understood ways. In the HealthNuts study, peanut allergy was more common in children with at least one parent born in East Asia than in those with parents born in Australia, with an odds ratio of 3.4 [46]. Keet et al. also demonstrated increased rates of food sensitization in US-born children of immigrant parents compared to the general population (odds ratio of 1.5); however, Asian was not a race included in the study [47]. Keet et al. hypothesized this may be due to an interaction between genetics: a naïve immune system primed to the ancestral milieu; the new environment: including differences such as decreased exposure to certain infections, especially helminths; and adoption of different dietary exposures [47].

**Fig. 1:**
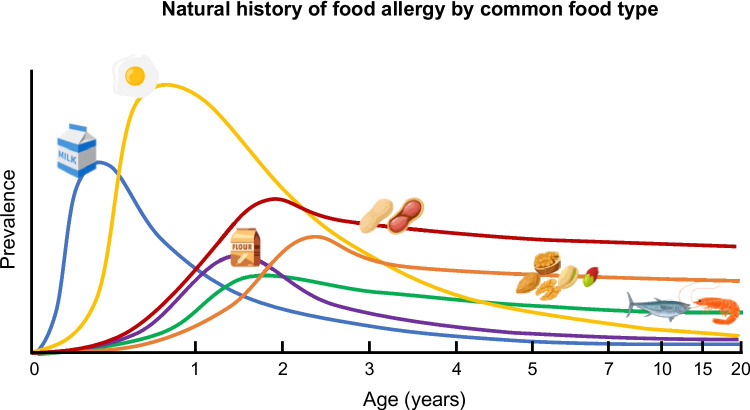
Infographic illustrating the relative prevalence of common food allergies at different ages to milk, egg, peanut, tree nuts, wheat, and seafood

Much like the trends observed in various allergic disorders that fluctuate with age (termed the atopic march), the age of onset, prevalence, and natural history of specific food allergies vary by food (Fig. 1). At present, most evidence is derived from retrospective cohort studies and there are few well-designed longitudinal studies prospectively assessing these characteristics of food allergies.

### Onset of Food Allergies in Infancy

IgE-mediated food allergy to milk, soy, egg, and wheat tend to develop in infancy, and typically resolve in childhood, while tree nut, fish, and shellfish allergies appear to develop slightly later and often persist into adulthood, perhaps a reflection of typical timing of food introduction to an infant’s diet. The onset of peanut allergy traditionally was thought to occur later, although there has been a rise in the prevalence of peanut allergy in infancy, possibly in relation to changing practices of earlier introduction of peanut [48].

Milk allergy is the most common food allergy with onset most commonly in infancy (< 12 months) [4]. Historically, there was a favorable prognosis for milk allergy with high rates of resolution [4]; however, recent studies have shown marked heterogeneity in rates of resolution across geographic regions (Table 1). It is difficult to compare studies given methodological differences (and in the case of Host et al., the inclusion of possible non-IgE-mediated milk allergy); however, these variations may reflect a more highly atopic population overall in recent years. Between 10 and 14% of infants with milk allergy also are sensitized to soy, with some having clinical IgE-mediated soy allergy [49]. Although soy allergy is not as common (estimated to affect 0.4% of children) [15], soy allergy presents at a similar age to milk allergy, with onset in early infancy. Few studies have examined the resolution of soy allergy (Table 1).

**Table 1:** Age of acquisition of tolerance for common food allergies

**Food**	**Tolerance acquisition**
**Milk**	[Europe] 60% by 12 months [4] [Denmark] 85–90% by 3 years [5] [US] 41% by 4 years [1] [US] 50% by 5 years [6] [Korea] 50% by 8–9 years [7] [Denmark] 87% by 3, 92% by 5, 97% by 26 years [8] [US] 64% by 12 years, 79% by 16 years [9]
**Egg**	[Europe] 50% by 1 year [10] [Australia] 47% by 2 years [11] [US] 50% by 3 years [12] [Japan] 59% by 5 years [13] [US] 37% by 10 years, 68% by 16 years [14]
**Soy**	50% by 7 years [15]
**Wheat**	20–29% by 4 years 52–56% by 8 years 65–66% by 12 years [16, 17]
**Peanut**	22% by 4 years [11] 29% by 6 years [18••]
**Tree nuts**	9 to 14% after 4 years (median age not specified) [19]
**Fish**	3.4–26% by 4–5 years [20, 21]
**Shellfish**	3.9% after 5–10 years [22]

Upon introduction of solids, egg allergy becomes one of the commonest IgE-mediated food allergies in infants. The prevalence varies across geographic regions, and much like milk allergy, egg allergy tends to resolve in early childhood (Table 1). Wheat allergy is the third commonest food allergy in preschool children, particularly in Europe and the US [50, 51]. Two studies (US and Poland) examining the natural history of wheat allergy showed similar rates of resolution, with a median rate of resolution of 5 to 6½ years of age (Table 1) [16, 17].

Various factors appear to predict the likelihood of resolution or persistence of infant-onset food allergy. Larger skin prick test (SPT) wheal size and higher serum specific IgE (sIgE) level predict lower rates of resolution, for example, an egg SPT > 4 mm or sIgE > 1.7 kU/L at 1 year of age was predictive of persistent egg allergy at 2 years of age (OR 3.32 and 29.46 respectively) [18••]. Similar trends are seen for milk, soy, and wheat, where higher peak sIgE and/or larger SPT predict persistent allergy [9, 15, 16]. Surprisingly, with the exception of milk allergy, where comorbid asthma and allergic rhinitis are significant predictors of persistence [9], the presence of atopic dermatitis or other atopic conditions has not been shown to predict rates of resolution of food allergies.

Certain interventions have the potential to alter the natural history of food allergy in infancy. There is now mounting evidence supporting early introduction of allergenic foods to prevent the development of food allergies [52, 53], consistent with the “dual allergen exposure” hypothesis. Milk and egg ladders to allow dietary inclusion of tolerated forms of milk and egg in children with these allergies may increase the rate of and accelerate the acquisition of tolerance. In studies where prospective cohorts of children who passed challenges to baked milk or baked egg and were instructed to continue daily consumption were compared with a retrospective comparison group (matched for age, sex, and baseline sIgE level) who were managed with strict milk or egg avoidance, and offered whole milk or egg challenges only as part of routine clinical care, the intention-to-treat groups were 6 times and 5 times more likely to become milk and egg tolerant, respectively. The active groups in these studies also became tolerant faster than the comparison group; 76% versus 33% at 60 months for milk; and 65% versus 15% at 60 months for egg [54, 55]. Despite these promising results, food ladders remain inconsistently implemented as a clinical tool, and thus there is a need to establish standardized protocols to ensure successful and safe utilization.

### Onset of Food Allergies in Early Childhood

The onset of peanut, tree nut, fish, and shellfish allergies tends to be later than milk, egg, soy, and wheat, with estimated peak prevalence at 1 ½ to 3 years of age [19, 21, 56, 57], although the shift towards early introduction of these foods has led to a corresponding reduction in age at diagnosis of these allergies. In addition to presenting later, nut and seafood allergies also tend to persist into adulthood, which will be covered more in the following sections (Table 1). Co-reactivity between peanut and tree nuts is common, ranging from 10 to 40% in certain populations [58, 59]. Although tree nut allergies are reportedly less prevalent than peanut allergy, tree nut allergies have a similar natural history profile, though with lower rates of resolution compared to peanut allergy (9–14% versus 22% at 4 years) (Table 1) [19]. Fish and shellfish are common causes of food allergies worldwide, with higher prevalence found in Asian and Northern European countries where seafood is more frequently consumed. Ages of onset for fish and shellfish allergy are approximately 1 ½ years and 5 years, respectively, with a wide range reported for rates of resolution (Table 1) [21, 60, 61]. Sesame is an emerging culprit for food allergy in childhood, with estimated prevalence in children of 0.2–0.8% [25, 62, 63]. Sesame allergy studies show slightly higher rates of resolution compared to peanut, tree nuts, fish, and shellfish, with most studies reporting an estimated 30% of children acquiring tolerance by 4–5 years [1, 62, 64].

### Considerations in Infants and Young Children with Food Allergy

Rates of food allergies, especially in infancy and early childhood, are at an unprecedented high. This is a critical age for interventions that may alter the natural course of food allergies. And although childhood food allergies to staple foods have generally favorable long-term prognoses, strictly avoiding these foods for months to years may have negative health, economic, and social consequences. As integral components of infant formula, and common early solids, omitting staple items such as milk, egg, and wheat can lead to growth restriction and nutritional deficiencies in calcium, vitamin A, and vitamin D [65]. There is also an increased cost burden for patients and their families living with food allergies: examples include out-of-hospital services, prescriptions, and nutrition products (including hypoallergenic formulas) [66, 67]. Additionally, parents or caregivers of food-allergic infants and children report higher rates of anxiety, and report food-allergy specific distress, and post-traumatic stress symptoms following anaphylactic reactions [68, 69], which unnecessarily limit participation in social activities, impact daycare attendance, trigger maladaptive coping and illness adaptation, and affect family dietary practices including for siblings, with overall compromise in health-related quality of life [70, 71]. Intervening at an early age is crucial to prevent such deleterious consequences. By understanding the natural history of food allergies, health professionals can counsel and educate parents and caregivers on expectations and prognosis. For foods with good prognosis, it is important to offer food challenges within an appropriate time frame to ascertain tolerance which then avoids unnecessary prolongation of food avoidance.

Lastly, there is a growing paradigm shift towards the implementation of oral immunotherapy (OIT), with infancy a specific target age group, especially in those who fail primary prevention and for food allergies that have a higher risk of persisting. Trials examining peanut OIT in infancy and preschool-age children included relatively few subjects but demonstrated a relatively favorable safety profile and good efficacy, with 81–91% tolerating 3–4 g of peanut protein at the end of treatment [72, 73]. There are fewer studies focusing on oral immunotherapy to milk and egg in children, and rates of adverse events are still high [74, 75]. Given the favorable prognosis, it is also difficult to differentiate the effect of milk and egg OIT from the natural course to the acquisition of tolerance. Real-world applicability of OIT in altering the natural course of food allergy has yet to be validated, with considerations of patient selection, feasibility, and safety still requiring further evaluation.

## Adolescence

Allergies can persist from childhood into adulthood, or they can resolve within the teenage years. Additionally, new food allergies can present for the first time during adolescence. Overall, the prevalence of food allergy in the adolescent age group is increasing, with studies identifying rates of 4–7.1% over the last decade compared to 1% two decades ago [76–79]. An Australian study identified the prevalence of clinician-diagnosed food allergy in early adolescence (10–14 years) to be 4.5% (compared to self-reported food allergy of 5.5%), with the most common allergens being peanut (2.7%) and tree nuts (2.3%) [77]. Venkataraman et al. reported a rise in food allergy prevalence in the UK to 4% at age 18 from 2.3% at age 10, likely due to new food allergy acquisition [78]. Gupta et al. found the overall reported food allergy prevalence for US adolescents 14–17 years to be 7.1% [79]. The rate of anaphylaxis due to food allergy in adolescents has also been increasing, with a 2.1- and 1.5-fold increase in anaphylaxis-related admissions to Australian hospitals in 5–14-year-old children and 15–29-year-old individuals respectively over a 5 year period, which may be due to this increasing prevalence [80].

### Tolerance and Persistence of Food Allergy in Adolescence

Milk allergy often resolves in childhood, but tolerance can also be achieved in early adolescence [81]. A study from the Isle of Wight found that milk allergy prevalence decreased to 0.3% at age 18 years from 0.5% at age 10 years, indicating continued resolution throughout late childhood and adolescence [78]. Skripak et al. reported that tolerance continues to develop during adolescence, showcasing the importance of continued evaluation during this period to facilitate the demonstration of tolerance (Table 1) [9]. This study also found that persistent milk allergy was more likely in patients with higher milk-specific IgE levels, especially those with peak levels ≥ 20 kU/L [9]. Another study found that patients at higher risk for persistent milk allergy were those with high milk-specific IgE, large milk SPT wheal size, and more severe eczema [6, 82].

Egg allergy can persist into the school-aged years, and occasionally even longer. Tolerance to egg almost doubles during the early years of adolescence, but there is a wide range of described rates of resolution depending on the study population (Table 1) [14, 81]. Factors correlated with prolonged egg allergy include high egg-specific IgE levels, male gender, diagnosis of atopic dermatitis at 1 year, sensitization to one other food, lower threshold for reaction, skin reaction with first oral food challenge, and baked egg allergy at 1 year [3, 18••].

Due to the resolution of milk and egg food allergies, the prevalences of peanut, tree nut, and shellfish allergies surpass those of milk and egg by early adolescence.

Peanut allergy most often persists into adulthood, but can resolve during childhood, or less often during adolescence. Gupta et al. found the prevalence of peanut allergy in early childhood, adolescence (14–17 years) and adulthood to be 2.6%, 2.1%, and 1.8%, respectively [2•, 79, 83]. Risk factors for peanut allergy persistence include diagnosis of atopic dermatitis at age one, sensitization to at least one tree nut/one other food/or dust mite, a low threshold for peanut reaction during an oral food challenge at age one [18••], peanut-specific IgE (sIgE) ≥ 1 kU/L at diagnosis [84], and anaphylaxis as initial reaction [85]. Having at least one Asian parent was a risk factor in both persisting egg and peanut allergy in the Australian HealthNuts study [11]. The reasons for this are unclear.

Tree nut allergy and shellfish allergy are each present in approximately 20% of adolescents with food allergy [86]. Tree nut allergy often persists into adolescence and adulthood, with Gupta et al. identifying a frequency of 0.9% of individuals 14–17 years with tree nut allergy in a cross-sectional survey of US households [79]. Children at risk of persistent tree nut allergy include those with elevated IgE levels, atopic dermatitis, or active allergy to a different food or another tree nut [58].

Fish allergy can develop in childhood and is often persistent [87]. However, studies have shown up to 26% of fish-allergic children can develop tolerance in adolescence (Table 1) [20, 21]. A recent study from Singapore also found that many children who have fish allergy can tolerate other fish species and have the possibility of fish allergy resolution [20], which highlights the importance of follow-up to evaluate fish species that can be tolerated and introduced into the diet, although this may be difficult as misidentification and mislabeling of fish is common [88]. There is also data to suggest that there is increasing development of fish allergy in later adolescence and adulthood, with a US study demonstrating the prevalence of fish allergy to be 0.2% at 6–17 years, and 0.5% at 18–40 years [89].

### Onset of Food Allergy in Adolescence

Shellfish allergy often arises in adolescence onwards and is usually persistent with prevalence varying significantly based on geographic region [87]. Adolescents (14–16 years) in the Philippines and Singapore have prevalence rates of patient/parent-reported shellfish allergy of around 5% versus 1.2% in Singaporean children 4–6 years [90]. In contrast, a US survey of individuals ages 6–17 years found a significantly lower shellfish allergy prevalence rate of 0.7% [89], likely owing to the relatively lower frequency of shellfish consumption in the US diet.

Peanut and tree nut allergies often develop in childhood; however, these allergies can also present in adolescence or adulthood as both IgE-mediated food allergy and the pollen-food allergy syndrome [81]. Venkataraman et al. identified the overall prevalence of peanut and tree nut allergies at 10 and 18 years of age, respectively, of 0.4% and 1.0% for peanut, and 0.2% and 0.5% for tree nuts, with the increase suggesting a proportion of individuals with new-onset peanut allergy in adolescence [78].

Older age is a risk factor for developing pollen-food allergy syndrome (PFAS)/oral allergy syndrome (OAS), a contact allergic reaction resulting only in oropharyngeal mucosal symptoms after ingestion of certain fruits and vegetables. PFAS/OAS arises from cross-reactivity between pollen and raw plant-derived food allergens in pollen-allergic individuals. The prevalence varies by region depending on pollen exposure [91]. PFAS/OAS can present in childhood, adolescence, or adulthood with prevalence rates ranging from 4.7 to over 20% in children and from 13 to 58% in adults, suggesting increased development over a lifetime and that PFAS/OAS may often be persistent [92]. Yasudo et al. found that childhood symptoms of wheeze, eczema, or allergic rhinitis at age 5 or 9 were risk factors for the development of PFAS/OAS by age 13 years [93].

### Considerations for Adolescents with Food Allergy

Adolescents and young adults are thought to be at a higher risk of severe or fatal food-induced reactions, often attributed to delayed administration of epinephrine [94]. This may occur due to reluctance, denial about the severity of a reaction, or lack of availability of epinephrine. Adolescence is a time when some may engage in risk-taking behaviors. Sampson et al. found that only 61% of surveyed US teenagers always carry their EpiPens, and overall, the frequency with which adolescents carry their EpiPens differed based on their activity, with sports being the lowest at 43% [95]. A “high-risk” group of individuals (17% of the sample) who did not always carry epinephrine and would knowingly ingest foods that they had been told to regard as risky was identified; this group reported feeling “different” from their peers due to their food allergy diagnosis [95]. This study highlights the importance of educating teenagers and their guardians, as well as peers of those with food allergy, to further normalize food allergy safety practices.

It is also important to consider the mental health effects that a food allergy diagnosis has on the adolescent age group. A recent systematic review analyzed the burden of food allergy on teenagers' quality of life and found overall decreased health-related quality of life, largely due to concerns about public allergen exposures, social limitations due to food allergy, and bullying or teasing [96]. Only one of the studies included in this review directly compares health-related quality of life between children (0–12 years) and adolescents (13–17 years) [97]. This paper suggests that there is worsening health-related quality of life with age among individuals with food allergy; however, this may be confounded by the fact that childhood measurements are proxy reports provided by parents [97]. This highlights the importance of assessing the mental health status of adolescents with food allergies. Rubeiz et al. report that allergists can empower patients with food allergy education to enhance resilience in this population [98]. All physicians play an important role in addressing mental health for adolescents, especially for those with chronic health conditions such as food allergy.

Adolescents should continue to be evaluated by allergists to evaluate the potential to develop tolerance. Especially as teenagers prepare to leave home, whether to college or otherwise, it becomes important for them to have clarity surrounding a food allergy diagnosis and which foods they can safely eat versus must avoid completely. Similarly, as adolescents begin to gain independence in many areas of their lives, they also take ownership of their medical conditions, such as food allergies. Conversations with adolescents should empower them to develop good risk management practices (such as always clearly declaring their allergens when eating out and always reading labels), understand their allergy action plan, and be familiar with using emergency medications to ensure that they can safely care for themselves.

## Adulthood

The prevalence of adult food allergy is at a historic high [79]. The most common allergies reported in adults are shellfish (2.9%), milk (1.9%), peanut (1.8%), tree nut (1.2%), finned fish (0.9%), egg (0.8%), wheat (0.8%), and sesame (0.2%) [2•, 63]. The population of food-allergic adults is comprised of two, often overlapping, categories: those with persistent childhood allergies, and those with new-onset allergies in adulthood. However, there is a relative scarcity of information available regarding the natural history of food allergy in adults in comparison to children and adolescents.

While food allergies in adulthood are common, the proportion of adults who believe themselves to be food allergic is even higher. In a population-based survey of 40,443 adults aged 18 and older, nearly 19% of respondents believed they were food allergic; however, only 10.8% of those adults reported symptoms convincing for IgE-mediated food allergy, while an additional 8.2% reported symptoms inconsistent with IgE-mediated food allergy [2•]. The overlap of food allergy symptoms with PFAS/OAS may play a role in this discrepancy, as PFAS/OAS is the most common food allergy in adults, estimated to affect 13–58% of adults in various studies [92].

Often, adults continue to practice strict avoidance of culprit foods based on the assumption that food allergy is lifelong [99]. However, recently, there has been increased interest in challenging this assumption. One study of 35 adults with a clinician-confirmed history of peanut allergy found that up to 20% tolerated peanut at double-blind placebo-controlled food challenges [99]. As our understanding of allergy acquisition and tolerance in adulthood increases, so too does our ability to reassess long-standing food allergy labels.

### Persistent Childhood-Onset Food Allergies

A significant proportion of children with food allergies will acquire tolerance over time while certain foods including peanuts, tree nuts, fish, and shellfish are known to frequently persist into adulthood [3, 86]. For these foods, several factors are predictive of allergy persistence into adulthood, including earlier age of diagnosis and the presence of comorbid atopic disorders such as allergic rhinitis, asthma, and atopic dermatitis [100, 101]. Symptom severity on ingestion and lower threshold for reaction are both associated with allergies that are the most likely to persist [3]. In a US Food Allergy Research & Education (FARE) survey, among adults who reported childhood-onset food allergy, natural tolerance acquired at an unspecified age was most frequently reported to egg (38%), meat (30%), and milk (26%) [102••].

Larger SPT wheal size and higher levels of food-specific IgE are also highly associated with persistent allergy [3]. Among individuals with a milk-specific IgE over 50 kU/L, 40% will remain milk-allergic into adulthood [9]. Similarly, only 11% of children with egg IgE ≥ 50 kU/L will outgrow their allergy by 18 years [14]. Peanut allergy is less likely to be outgrown than allergies to milk and egg, though lower peanut IgE and peanut SPT are associated with greater likelihood of outgrowing this allergy [99].

### Adult-Onset Food Allergies

While de novo food allergies have classically been viewed as a pediatric concern, food allergies can develop at any age. The phenomenon of adult-onset food allergies has gained increasing recognition in recent years, with a growing body of research examining the etiology, presentation, and natural course of adult-onset food allergies [86]. One retrospective study found that 15% of all documented food allergies had onset in adulthood [101]. In contrast, in a cross-sectional survey study, 48% of all food-allergic adults reported developing at least one of their food allergies in adulthood, and over a quarter of food-allergic adults reported developing allergies only in adulthood [2•]. There is a wide age range encompassing age of first reaction in adult-onset food allergy, though a peak appears in the early 30s (mean 31, range 18–86) [101]. In contrast to the male predominance seen in childhood-onset food allergy, adult-onset food allergy has a female predominance of 64% [101].

A key difference between the foods responsible for allergy in adults compared to children and adolescents is the increased prevalence of allergies to shellfish and fish. In addition, in US adults there are high rates of incidence of IgE-mediated allergy to wheat (52.6%) shellfish (48.2%), soy (45.4%), and finned fish (39.9%) [2•]. It has been speculated that one potential reason for the different foods responsible involves the frequency of ingestion, as many adults will report a period without recent ingestion of a previously tolerated food prior to the development of allergy [101]. In the same FARE survey referenced above, among those with adult-onset food allergy, tolerance was most commonly reported to tree nuts (13%), egg (11%), and fruits (10%) [102••].

### Considerations in Adult Food Allergy

The food-allergic adult requires several age-specific considerations, as adults are less likely to have regular follow-up with an allergist, or to have an active emergency action plan. In general, adults tend to experience food-allergic reactions that are more systemic than in children [103]; contributing factors may include delayed administration of epinephrine and higher rates of comorbidities. Over half of food-allergic adults report a history of at least one severe reaction, and 38% of adults reported at least one food allergy-related emergency department visit in their lifetime [2•]. However, only 15–24% of food-allergic adults reported receiving or carrying a current epinephrine prescription [2•, 23].

One reason for the increased severity of reactions in adults may be increased sensitivity to the effects of cofactors [104]. Medications including nonsteroidal anti-inflammatory drugs, beta blockers, and antacids, which are more likely to be taken by adults, can impact both the severity and management of allergic reactions [103]. Similarly, alcohol consumption can both increase the severity of allergic reactions, as well as the potential likelihood of a reaction occurring due to decreased attentiveness or caution. Hormonal fluctuations, such as menstruation and menopause, can also play a role in both the development and manifestations of allergic diseases [105]. Finally, adults are also highly susceptible to the effects of exercise and often exhibit coupling of food reactions and exercise, as with wheat-dependent exercise-induced anaphylaxis [103]. Given these many potential exacerbating factors, food allergy education tailored to the adult population is critical to prevent food allergy-related morbidity and mortality.

Disparities also remain in the clinical management of adult food allergies. Given that most studies evaluating food immunotherapy to date have occurred in children, treatment options and interventions tend to be more limited for adults [103]. Palforzia, a treatment for peanut allergy, and the only FDA-approved food immunotherapy, is approved for children aged 4 to 17 [103]. There are often significant barriers to recruiting adults to food allergy trials, both due to logistical challenges (i.e., family or work commitments) and decreased interest secondary to the normalization of food allergy in one’s life as a chronic disorder. However, given the increasing rates of adult food allergies, additional research on potential treatment strategies is needed.

## Conclusions

There has been an unprecedented rise in food allergies worldwide in recent decades, with the cause of this epidemic remaining unclear. New risk factors have been identified for the development of food allergy, particularly risk factors underpinning the dual allergen hypothesis. Changes have also been observed in the natural history of food allergy in that allergies to certain foods thought previously to resolve early are now persisting into adulthood, and there is an increased prevalence of adolescent and adult-onset food allergies. On the other hand, there is new recognition of the potential to outgrow food allergies in adulthood which were previously thought to be lifelong. It is important to stay up to date with these developments as this can provide diagnostic and prognostic information and helps guide clinicians in their management of individuals with food allergies. Furthermore, better understanding of the current state of food allergies, rates of acquisition of tolerance, and predisposing factors for persistent allergy will facilitate the ongoing development of new therapies to alter the course of natural history.

## Data Availability

Our paper is a literature review, not original research, and so the only data that it relies upon are the papers listed in the references.
